# Cytogenomic Abnormalities in 19 Cases of Salivary Gland Tumors of Parotid Gland Origin

**DOI:** 10.1155/2020/8897541

**Published:** 2020-12-02

**Authors:** Marie Zerjav, Autumn DiAdamo, Brittany Grommisch, Amato Katherine, Hongyan Chai, Gang Peng, Peining Li

**Affiliations:** ^1^Department of Genetics, Yale University School of Medicine, New Haven, CT, USA; ^2^Diagnostic Genetics Sciences Program, University of Connecticut, Storrs, CT, USA; ^3^Department of Biostatistics, Yale School of Public Health, New Haven, CT, USA

## Abstract

Salivary gland tumors (SGTs) of parotid origin are a group of diverse neoplasms which are difficult to classify due to their rarity and similar morphologic patterns. Chromosome analysis can detect clonal abnormalities, and array comparative genomic hybridization (aCGH) analysis can define copy number alterations (CNAs) from tumor specimens. Of the 19 cases of various types of SGTs submitted for cytogenomic analyses, an abnormal clone was detected in nine cases (47%), and CNAs were detected in 14 cases (74%). Recurrent rearrangements involving the PLAG1 gene at 8q12, recurrent CNAs including deletions of 6q, 9p (CDKN2A), and 17p (TP53), loss of Y chromosome, and gain of chromosome 7 were defined from these cases. Combined karyotyping and aCGH analyses could improve diagnostic yield. Future study for more precisive correlation of SGT classification with cytogenomic abnormalities will facilitate better diagnosis and treatment.

## 1. Introduction

The salivary glands comprise three major paired parotid, submandibular, and sublingual glands and minor glands located in the palate, lips, and buccal mucosa; salivary gland tumors (SGTs) are rare neoplasms accounting for 0.4–13.5 cases per 100,000 people [1]. The 2005 World Health Organization (WHO) classified SGTs into ten benign and 23 malignant entities of epithelial tumors, and more recent 2017 WHO classification listed new entities and key genomic alterations [1, 2].

Earlier cytogenetic analysis has aided in the diagnosis of salivary gland tumors [3–6]. Recurrent chromosomal abnormalities such as a deletion of the long arm of chromosome 6, gain of an extra copy of chromosomes 7 and 8, translocations involving 8q12, and a loss of Y chromosome have been detected in certain types of SGTs. Comparative genomic hybridization (CGH) using fluorescently labelled tumor and reference DNAs cohybridized to normal metaphases was introduced to analyze chromosomal imbalances from paraffin-embedded SGTs [7, 8]. Several studies using high-resolution array CGH (aCGH) to detect genomic copy number alterations (CNAs) and to further map candidate oncogenes and tumor suppressor genes in SGTs have been reported [9–14]. The use of cytogenetics for diagnosis of SGT required an accurate correlation of recurrent clonal abnormalities with tumor classification. There are some tumor types that fall into clear cytogenetic categories and several tumors that contain multiple diverse anomalies, while others have an apparently normal karyotype. In these instances, it may be beneficial to supplement karyotyping with other techniques. The use of aCGH can define genomic coordinates and gene content of visible chromosomal imbalances and cryptic aberrations that otherwise may not be visible through chromosome analysis. In this study, we performed combined karyotyping and aCGH analyses on 19 cases of SGTs to further evaluate the technical utility and clinical significance.

## 2. Clinical Findings and Cytogenomic Results

After parotidectomy, tumor specimens from 19 cases of various types of SGT of parotid origin were collected and submitted for pathology and cytogenomic analyses. Cell culture set-up, metaphase preparation, and karyotyping were performed on metaphase spreads from these tumors using laboratory's standardized procedures [15]. DNA was extracted from the tumor specimens using the Gentra Puregene Kit (Qiagen). aCGH analysis was performed on SurePrint G3 Human CGH 8 × 60 K Microarray Kit (Agilent Technologies) as previously described [16, 17].

The pathologic findings for these cases included six specimens of pleomorphic adenoma (PA), one specimen each for basal cell adenoma (BCA), Warthin tumor (WT), oncocytoma, salivary duct carcinoma (SDC), Hodgkin's lymphoma (HL), Merkel cell carcinoma (MCC), squamous cell carcinoma (SCC), and six specimens of unspecified SGTs. Karyotypic analysis detected clonal abnormalities in nine cases (47%), and aCGH analysis detected CNAs in 14 cases (74%). The genomic coordinates for these CNAs by human genome assembly NCBI36/hg18 (https://genome.ucsc.edu/) are listed in Supplemental Table 1. The age, gender, pathologic diagnosis, site of parotid, karyotypic results, and major aCGH findings from these 19 cases are summarized in Table 1.

**Figure 1:**
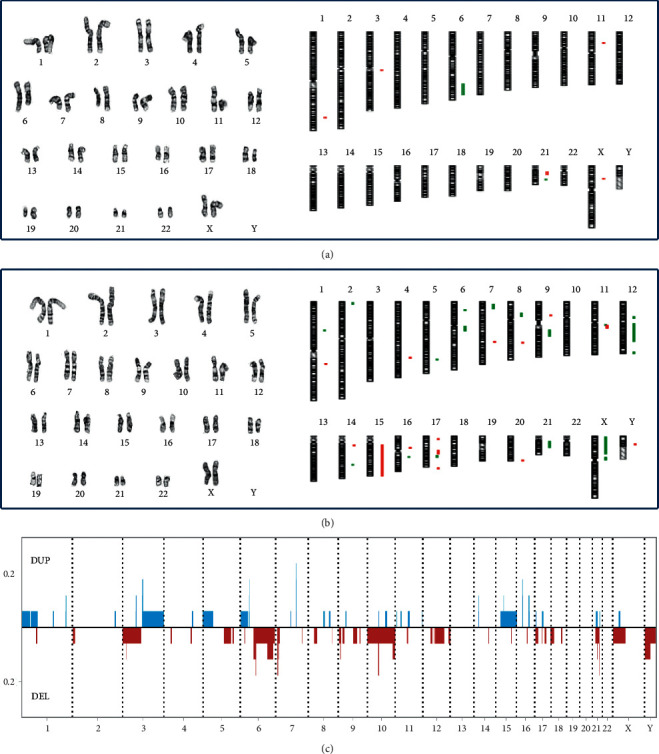
Cytogenomic abnormalities detected in 19 cases of SGTs. (a) For case 4, representative karyotype showed an abnormal clone with a deletion in the long arm of chromosome 6 and a paracentric inversion in the long arm of chromosome 8 (left); genome view by aCGH showed CNAs of deletions of 6q and 21q and duplications of 1q, 3q, 11p, 21q, and Xp (right, green bar for deletion and red bar for duplication). (b) For case 7, chromosome analysis showed a normal female karyotype (left); aCGH genome view showed numerous CNAs in multiple chromosomes (right). (c) The distribution of CNAs detected from 14 cases.

Cytogenomic abnormalities were detected in the six cases with PA. Case 1 had an abnormal clone featuring a recurrent translocation t (5;8)(p13;q12) likely with *LIFR*-*PLAG1* gene rearrangement causing PA; a normal result by aCGH indicated a balanced translocation. Case 2 had an abnormal clone with a pericentric inversion in chromosome 4, which was considered a balanced rearrangement from the normal result by aCGH. Case 3 had an abnormal clone with a ring chromosome, but aCGH showed a normal result; this abnormal clone may be in a low percentage of tissue specimen and below the detection threshold of aCGH. Case 4 had a stemline clone with an apparently paracentric inversion in the 8q12-q24 region and a sideline clone with an additional aberration of a deletion in 6q; aCGH defined a 29.182 Mb deletion of 6q23.1-q25.3 and six additional CNAs (Figure 1(a)). Case 5 had a normal male karyotype and CNAs of duplications of 10q21.1, 10q23.1-q23.3, and 21q22.11-q22.12 and deletions of 17p13.2-p13.1 and 17q21.2; the deletion at 17p13.2-p13.1 included the *TP53* gene. Case 6 had an abnormal clone with an insertion of 2p25.1-p21 segment into 12q13; aCGH detected CNAs of duplications of 2q33.2-q33.3, 3p14.1, 3q11.2, 16q21 and a deletion at 21q22.11.

Case 7 with BSA had a normal female karyotype but a gain of chromosome 15, a deletion of Xp, and numerous CNAs in multiple chromosomes by aCGH (Figure 1(b)). Case 8 with WT had a normal female karyotype but CNAs of duplications of 6p21.1, 7q22.1, and 16p11.2 and deletions of 7p21.3 and 18q21.2-q21.31. Case 9 with oncocytoma had an abnormal clone of a gain of chromosome 7; aCGH detected CNAs of duplications of 7q22.1, 16p13.3, and 16p11.2 and a deletion at 10q11.23-q21.1. Case 10 with SDC had a normal male karyotype; aCGH detected a loss of Y chromosome and additional CNAs at 4p, 8q, 11q, 15q, and 21q. Case 11 with HL had a normal female karyotype and a normal result by aCGH. Case 12 with MCC had a normal male karyotype; aCGH detected large segmental losses of 3p, 5p, and one copy of chromosome 10, a gain of 3q, and additional CNAs in multiple chromosomes. Case 13 with SCC had a normal male karyotype and a normal result by aCGH.

For six cases with unspecified SGT of parotid origin, three cases had an abnormal clone detected by chromosome analysis, and all had CNAs detected by aCGH. Case 14 had an abnormal hypotriploid clone with complex numerical and structural rearrangements; aCGH detected a 33.478 Mb deletion of 5q21.1-q31.1 and a 103.653 Mb deletion of 6q11.1-q27 and several CNAs of deletions of 9p22.1-p21.2 (*CDKN2A* gene), 9q31.1, 10q26.13-q26.3, 17p13.1-p12, and 18p11.32-p11.21. Case 15 had an abnormal clone with an apparently balanced translocation t (4;7)(q12;q22) and CNAs of duplications of 7q22.1 and 16p13.3 and a deletion at 10q11.23-q21.1. Case 16 had a stemline abnormal clone with a loss of Y chromosome and a sideline clone with an additional aberration of a gain of chromosome 7; a loss of Y chromosome was detected by aCGH. Cases 17–19 had a normal karyotype and a few CNAs detected by aCGH.

A genome view of these CNAs from the 14 cases was plotted to define the distribution and recurrence across the genome (Figure 1(c)). The overlapped regions from these CNAs could be constructed to identify recurrent segments for mapping candidate oncogenes and tumor suppressor genes. Excluding gains and losses of entire chromosomes or whole arms, a total of 90 CNAs were detected; 38 were recurrent CNAs in 15 chromosomal loci (Supplemental Table 2).

## 3. Discussion

aCGH analysis has been used extensively for prenatal and postnatal detection of constitutional copy number variants and showed significantly improved diagnostic efficacy and accuracy [19]. However, the application of aCGH to detect somatic CNAs in various cancers has been adapted slowly due to the difficulty in dissecting the clonal heterogeneity from CNAs and the deficiency of supporting databases to interpret abnormal findings [16, 17]. In this case series, the abnormality detection rate of 74% from aCGH was higher than the 47% from karyotyping. It is obvious that aCGH has the advantages to detect cryptic CNAs undetectable by karyotyping and to define the genomic coordinates and gene content from CNAs. However, several technical issues for combined karyotyping and aCGH have been noted. First, aCGH cannot detect balanced rearrangement, as seen in cases 1, 2, 4, and 15. Second, abnormal stemline and sideline clones detected by karyotyping could be missed by aCGH, as seen in cases 3 and 16. This is likely due to the analytical cutoff on detecting a mosaic pattern in 30% or less by aCGH [20]. Third, discordant results between normal chromosome and multiple CNAs raised concerns about the low mitotic index of tumor cells under in vitro cell culture. Despite these technical limitations, the integration of aCGH certainly showed improvement in defining tumor heterogeneity and genomic imbalances for identifying candidate tumor-related genes [18].

Of the six cases with PA, case 1 showed a recurrent t (5;8)(p13;q12) with *LIFR*-*PLAG* gene fusion known to cause PA, case 3 had a ring chromosome possibly from r (8)(p12q12.1) with *FGFR1*-*PLAG1* gene fusion, case 4 had an inv (8) (q12q24) possibly involving *PLAG1* gene rearrangement at 8q12, case 5 had a deletion at 17p including the *TP53* gene, and case 6 had an ins (12;2)(q13;p25.1p21) possibly involving rearrangement of *HMGA2* and *MDM2* genes of 12q13q15. Fluorescence in situ hybridization (FISH) using a panel of probes for these genes could be very helpful in detecting these recurrent rearrangements [21, 22]. The deletions of 6q showed two overlapped regions of 6q13 and 6q23.1q25.3 in cases 4, 7, 14, and 19. Acquired deletions of 6q were recurrent CNAs recognized in several types of SGTs from previous studies [4, 6–8], and two critical regions at 6q23.2 and 6q27 were specified [6]. Loss of Y chromosome in cases 10 and 16 and gain of an extra chromosome 7 in cases 9 and 16 were also noted in previous studies [4, 5]. A deletion at 9p22.1p21.2 including the *CDKN2A* gene in case 14 was a known recurrent CNA in multiple types of tumors. From the recurrent CNAs (Supplemental Table 2), a 3 Mb deletion at 10q11.23q21.2 was detected in cases 9 and 15, which likely involved the *PRKG1*-*MBL2*-*PCDH15* genes and truncated the *PRKG1* and *PCDH15* genes, but its clinical significance is unclear; a 2 Mb deletion at 1q41 was detected in cases 4 and 12, which involved the *USH2A*-*ESRRG*-*GPATCH2*-*SPATA17*-*RRP15*-*TGFB2* genes and truncated the *USH2A* and *TGFB2* genes. Disruption of the TGFB/SMAD pathway has been implicated in a variety of human cancers.

In conclusion, cytogenomic analysis on these 19 cases of SGTs detected clonal chromosomal rearrangements and CNAs in 14 cases. Recurrent chromosome rearrangements and CNAs likely associated with SGTs were defined through a comparison of findings from previous studies. Further integration of karyotyping, FISH, and aCGH could improve the diagnostic accuracy and result interpretation. However, the presence of heterogeneous cytogenomic abnormalities and the lack of supporting evidence from expert consensus or bioinformatics databases have made it difficult to correlate some of these results to SGTs, especially the unspecified SCTs. Future studies on a large case cohort with well-classified SGTs will be helpful to establish a precisive correlation between cytogenomic abnormalities and clinical classification.

## Figures and Tables

**Table 1:** Cytogenomic abnormalities detected in the nineteen cases.

Case no.	Gender	Age (years)	Tumor classification	Karyotyping results	aCGH results (NCBI36/hg18)	Likely causal/recurrent CNAs
1	F	29	Pleomorphic adenoma	46, XX, t (5;8)(p13;q12) [10]	NL	LIFR-PLAG1 gene fusion
2	F	52	Pleomorphic adenoma, right parotid	46, XX, inv (4)(p11;q25) [15]	NL	—
3	M	44	Pleomorphic adenoma, right deep parotid lobe	47, XY, +*r* [10]/46, XY [5]	NL	Likely r (8) (p12q12.1) of FGFR1-PLAG1 fusion
4	F	75	Plemomorphic adenoma, right parotid	46, XX, inv (8) (q12q24) [6]/46, idem, del (6) (q22q25) [7]/46, XX [2]	A 29.182 Mb del of 6q23.1-q25.3, and CNAs of 1q, 3q, 11q, 21q, and Xp	Likely rearrangement with 8q12 (PLAG1), del 6q
5	M	28	Plemomorphic adenoma, left parotid	46, XY [18]	CNAs of 10q, 17p, 17q and 21q	Del 17p (TP53)
6	M	47	Plemomorphic adenoma, right parotid	46, XY, ins (12;2)(q13;p25.1p21) [15]	CNAs of 2q, 3p, 3q, 16q and 21q	Likely rearrangement of 12q13q15 (HMGA2, MDM2)
7	F	48	Basal cell adenoma, left parotid	46, XX [15]	Gain of 15, a deletion of Xp, and numerous CNAs in multiple chromosomes	Del 6q, dup 17p13.1 (TP53)
8	F	76	Warthin's tumor, left parotid	46, XX [18]	CNAs of 6p, 7p, 7q, 16p, and 18q	—
9	F	69	Oncocytoma, right parotid	47, XX,+7 [5]/46, XX [10]	CNAs of 7q, 10q, and 16p	+7
10	M	65	Salivary duct carcinoma, right parotid	46, XY [18]	Loss of Y and CNAs of 4q, 8q, 11q, 15q, and 21q	Loss Y
11	F	49	Hodgkin's lymphoma, right parotid	46, XX [18]	NL	—
12	M	42	Merkel cell carcinoma, right parotid	46, XY [18]	Losses of 3p and 10, gains of 3q and 5p, and CNAs in multiple chromosomes	—
13	M	84	Squamous cell carcinoma, right parotid	46, XY [18]	NL	—
14	F	84	Undifferentiated, parotid	61∼66<3n->, XXX, +X, +1, +del (1) (p31), del (1) (q31), i (1) (p10), i (1) (q10),−2, del (2) (p13), del (3) (p13), +4x2, del (4) (q31), add (4) (p15), del (5) (q31), i (6) (p10), del (6) (q21),−7, add (7) (p22), −8, add (9) (p22), −10x2, +11, del (11) (q23) x2, −12, add (12) (p13), add (14) (p11),−15x2, −17, add (17) (p13), −18, −19, add (19) (p13),−21x2, +4∼8mar [cp6]	Large deletions of 5q and 6q, and CNAs of 9p, 9q, 10q, 17p, and 18p	Del 6q, del 9p (CDKN2A)
15	M	62	Parotid mass, lymphoma	46, XY, t (4;7)(q12;q22) [5]/46, XY [10]	CNAs of 7p, 7q, 9p, and 10q	—
16	M	77	Right parotid mass	45, X, −Y [3]/46, X, −Y, +7 [3]/46, XY [9]	Loss of Y	Loss Y, +7
17	F	69	Salivary, basal cells vs. adenoid	46, XX [15]	CNA of 3p	—
18	M	80	Parotid	46, XY [18]	CNAs of 8q	—
19	F	46	Left parotid gland	46, XX [18]	CNAs of 3p, 6q, 7q, 16q, and 21q	Del 6q

NL: normal; CNAs: copy number alterations.

## Data Availability

All data are included in the text and supplemental materials

## Supplementary Materials

Supplementary Materials Supplemental Table 1: genomic CNAs detected in 19 cases of SGTs of parotid origin. Supplemental Table 2: sorted overlapped regions for recurrent CNAs and possible candidate genes.
