# Supplementary material for: Cytogenomic Abnormalities in 19 Cases of Salivary Gland Tumors of Parotid Gland Origin
Source: Case Rep Genet. 2020 Dec 2;2020:8897541. doi: 10.1155/2020/8897541 (PMC7725583; doi:10.1155/2020/8897541)
Supplement: Supplementary Materials — Supplemental Table 1: genomic CNAs detected in 19 cases of SGTs of parotid origin. Supplemental Table 2: sorted overlapped regions for recurrent CNAs and possible candidate genes. [file 8897541.f1.zip › 8897541.f1/Suppl Tables 1_SGT_v5.pdf]

Supplemental Table 1. Genomic CNAs detected in 19 cases of SGTs of parotid origin*

Case No.	Regions	Genomic coordinates (hg18)	Size (Mb)	CNAs	Whole-arm/chr abns
4	1q41	chr1:213927297-216681725	2.754	dup	
	3q11.2-q12.1	chr3:95329130-99993678	4.665	dup	
	6q23.1-q25.3	chr6:130409579-159591681	29.182	del	
	11p14.2-p14.1	chr11:26677338-28306429	1.629	dup	
	21q21.1-q21.2	chr21:16373344-24560645	8.187	dup	
	21q22.11	chr21:33246146-34157533	0.911	del	
	Xp21.1	chrX:31996653-36210788	4.214	dup	
5	10q21.1	chr10:55040378-56196587	1.156	dup	
	10q23.1-q23.33	chr10:86199272-95156126	8.957	dup	
	17p13.2-p13.1	chr17:4462922-8216238	3.753	del	
	17q21.2	chr17:36740644-36963832	0.223	del	
	21q22.11-q22.12	chr21:34632707-34997218	0.365	dup	
6	2q33.2-q33.3	chr2:205367575-207460978	2.093	dup	
	3p14.1	chr3:64185680-66953491	2.768	dup	
	3q11.2	chr3:96210835-99173162	2.962	dup	
	16q21	chr16:58897364-63579800	4.682	dup	
	21q22.11	chr21:33246146-33982696	0.737	del	
7	1p31.1	chr1:72058394-76393250	4.335	del	
	1q22-q23.1	chr1:154140183-156831970	2.692	dup	
	2p25.3-p25.1	chr2:3055334-9137503	6.082	del	
	4q28.3-q31.1	chr4:138727543-141177344	2.45	dup	
	5q32	chr5:143289410-146866603	3.577	del	
	6p22.3	chr6:20434423-22592845	2.158	del	
	6q11.1-q14.1	chr6:62854106-76024836	13.171	del	
	7p21.3-p15.3	chr7:8489818-20689832	12.2	del	
	7q22.1	chr7:98708511-100967327	2.259	dup	
	8p12-p11.21	chr8:30336607-40456730	10.12	del	
	8p22.3	chr8:101947780-104198166	2.25	dup	
	9p13.3-p13.2	chr9:33900270-36589798	2.69	dup	
	9q21.11-q21.33	chr9:71840721-89218488	17.378	del	
	11q12.1-q12.2	chr11:57984015-60454740	2.471	del	
	11q12.2-q13.2	chr11:60478523-68454641	7.976	dup	
	12q12-q13.11	chr12:38765904-45595565	6.83	del	
	12q13.2-q13.3	chr12:54901971-56004927	1.103	del	
	12q14.1-q23.3	chr12:58369317-102792445	44.423	del	
	12q24.32-q24.33	chr12:125717085-131801952	6.085	del	
	14q11.2-q12	chr14:23237389-25075872	1.838	dup	
	14q24.2-q24.3	chr14:71443780-73860367	2.417	del	
	15q11.2-q26.3	chr15:23027575-102241547	77.311	gain	15q+
	16p11.2	chr16:29976404-30954138	0.978	dup	
	16q12.2	chr16:51371871-53914036	2.542	del	
	17p13.1	chr17:7081635-8151448	1.07	dup	
	17q21.2-q21.32	chr17:36472975-42191036	5.718	dup	
	17q22-q23.1	chr17:49284019-55252314	5.968	del	
	20q13.33	chr20:60881976-62634966	1.554	dup	
	21q11.2-q22.11	chr21:14640192-32123332	17.483	del	
	Xp22.33-q11.1	chrX:3059247-62858884	59.8	loss	Xp-

8	6p21.1	chr6:42141477-43419698	1.278	dup	
	7p21.3	chr7:8217664-12448273	4.23	del	
	7q22.1	chr7:99222667-102448818	3.226	dup	
	16p11.2	chr16:30002992-31504773	1.502	dup	
	18q21.2-q21.31	chr18:50001399-54362080	4.361	del	
9	7q22.1	chr7:99222667-102448818	3.226	dup	
	10q11.23-q21.1	chr10:52734291-56196587	3.462	del	
	16p13.3	chr16:1940255-3141763	1.202	dup	
	16p11.2	chr16:29983228-31504773	1.521	dup	
10	4p15.1-p14	chr4:33783065-37078326	3.295	del	
	8q23.3	chr8:114075883-115500013	1.424	del	
	8q24.22	chr8:133906398-134354287	0.448	dup	
	11q24.3	chr11:129287143-130089614	0.802	dup	
	15q15.1	chr15:38117022-39129686	1.013	dup	
	15q25.1	chr15:76621644-77108705	0.487	del	
	21q21.2-q21.3	chr21:23733501-26368243	2.635	del	
	Yp11.31-q12	chrY:2716261-57441009	54.725	loss	Y-
12	1p36.33-p35.1	chr1:749425-32472119	31.723	dup	
	1p34.3-p34.2	chr1:37039102-40651468	3.612	amp	
	1p34.1-p31.1	chr1:45468271-77578959	32.111	dup	
	1q41	chr1:214532032-216654201	2.122	dup	
	3p26.3-p11.1	chr3:134511-90336893	90.202	loss	3p-
	3q11.2-q29	chr3:95136354-199324877	104.189	gain	3q+
	4q28.2-q28.3	chr4:131095257-136998946	5.904	del	
	5p15.33-p11	chr5:204537-46136265	45.932	gain	5p+
	6p25.3-p21.31	chr6:1786486-36035503	34.249	dup	
	7p21.2-p21.1	chr7:14768534-16127866	1.359	del	
	7q11.23	chr7:72529024-75977418	3.448	dup	
	9p24.2-p23	chr9:2941153-9748013	6.807	del	
	10p15.3-q26.3	chr10:238264-135254661	135.016	loss	10-
	11p15.4	chr11:6211203-6596014	0.385	dup	
	14q11.2-q12	chr14:22307229-24112769	1.806	dup	
14	5q21.1-q31.1	chr5:101301623-134780095	33.478	del	
	6q11.1-q27	chr6:62796573-166449705	103.653	del	6q-
	9p22.1-p21.2	chr9:19597033-26999800	7.403	del	
	9q31.1	chr9:102946923-104087391	1.14	del	
	10q26.13-q26.3	chr10:123208606-133915151	10.707	del	
	17p13.1-p12	chr17:9173836-15133286	5.959	del	
	18p11.32-p11.21	chr18:14116-13762450	13.748	del	
15	7p21.3	chr7:8217664-12534438	4.317	del	
	7q22.1	chr7:99809498-101343821	1.534	dup	
	9p24.1-p23	chr9:8467034-12196342	3.729	del	
	10q11.23-q21.1	chr10:52888130-56196587	3.308	del	
16	Yp11.31-q12	chrY:2656261-28767745	26.111	loss	Y-
17	3p14.1	chr3:63858941-66953491	3.095	dup	
18	8q21.11-q21.12	chr8:75177077-78534818	3.358	dup	
	8q22.3-q23.1	chr8:105229117-107465156	2.236	dup	
19	3p24.3	chr3:15780938-16483557	0.703	del	
	6q13	chr6:74206488-75616871	1.41	del	
	7q32.2-q32.3	chr7:130068191-130771678	0.704	del	

16q21	chr16:58805277-63136942	4.332	dup
21q22.11	chr21:33411320-33982696	0.571	del

* A total of 90 CNAs and 9 whole-arm or whole chromosome abnormalities (highlighted).
