# Supplementary material for: Cytogenomic Abnormalities in 19 Cases of Salivary Gland Tumors of Parotid Gland Origin
Source: Case Rep Genet. 2020 Dec 2;2020:8897541. doi: 10.1155/2020/8897541 (PMC7725583; doi:10.1155/2020/8897541)
Supplement: Supplementary Materials — Supplemental Table 1: genomic CNAs detected in 19 cases of SGTs of parotid origin. Supplemental Table 2: sorted overlapped regions for recurrent CNAs and possible candidate genes. [file 8897541.f1.zip › 8897541.f1/Suppl Tables 2_SGT_v5.pdf]

Supplemental Table 2. Sorted overlapped regions for recurrent CNAs and possible candidate genes*

Case No.	Region	hg18	Size (Mb)	CNAs	Tumor Genes
9	10q11.23-q21.1	chr10:52734291-56196587	3.462	del	/PRKG1-MBL2-PCDH15/
15	10q11.23-q21.1	chr10:52888130-56196587	3.308	del	/PRKG1-MBL2-PCDH15/
7	14q11.2-q12	chr14:23237389-25075872	1.838	dup	
12	14q11.2-q12	chr14:22307229-24112769	1.806	dup	
7	16p11.2	chr16:29976404-30954138	0.978	dup	
8	16p11.2	chr16:30002992-31504773	1.502	dup	
9	16p11.2	chr16:29983228-31504773	1.521	dup	
6	16q21	chr16:58897364-63579800	4.682	dup	CDH8, CDH11/
19	16q21	chr16:58805277-63136942	4.332	dup	CDH8
7	17p13.1	chr17:7081635-8151448	1.07	dup	TP53
5	17p13.2-p13.1	chr17:4462922-8216238	3.753	del	TP53
7	1p31.1	chr1:72058394-76393250	4.335	del	
12	1p34.1-p31.1	chr1:45468271-77578959	32.111	dup	
4	1q41	chr1:213927297-216681725	2.754	dup	/USH2A-ESRRG-GPATCH2-SPATA17-RRP15-TGFB2/
12	1q41	chr1:214532032-216654201	2.122	dup	/USH2A-ESRRG-GPATCH2-SPATA17-RRP15-TGFB2/
7	21q11.2-q22.11	chr21:14640192-32123332	17.483	del	
10	21q21.2-q21.3	chr21:23733501-26368243	2.635	del	MRPL39, JAM2, ATP5J, APP
4	21q22.11	chr21:33246146-34157533	0.911	del	
6	21q22.11	chr21:33246146-33982696	0.737	del	
19	21q22.11	chr21:33411320-33982696	0.571	del	
6	3p14.1	chr3:64185680-66953491	2.768	dup	FRICKLE2, ADAMTS9, MAGI1, ALC25A26, LIRG1
17	3p14.1	chr3:63858941-66953491	3.095	dup	
6	3q11.2	chr3:96210835-99173162	2.962	dup	MTHFD2P, EPHA6, ARL6
4	3q11.2-q12.1	chr3:95329130-99993678	4.665	dup	
7	6q11.1-q14.1	chr6:62854106-76024836	13.171	del	
14	6q11.1-q27	chr6:62796573-166449705	103.653	del	
19	6q13	chr6:74206488-75616871	1.41	del	MTO1, EEF1A1, CD109,
4	6q23.1-q25.3	chr6:130409579-159591681	29.182	del	MYB 6q23.3-135.5 Mb
12	7p21.2-p21.1	chr7:14768534-16127866	1.359	del	
8	7p21.3	chr7:8217664-12448273	4.23	del	ICA1, NXPH1, FER4, NDUFA4, PHF14, THSD7A, TMEM106B
15	7p21.3	chr7:8217664-12534438	4.317	del	
7	7p21.3-p15.3	chr7:8489818-20689832	12.2	del	
7	7q22.1	chr7:98708511-100967327	2.259	dup	
8	7q22.1	chr7:99222667-102448818	3.226	dup	
9	7q22.1	chr7:99222667-102448818	3.226	dup	
15	7q22.1	chr7:99809498-101343821	1.534	dup	PILRA,.., CUX1
15	9p24.1-p23	chr9:8467034-12196342	3.729	del	PTPRD
12	9p24.2-p23	chr9:2941153-9748013	6.807	del	

*A total of 38 CNAs at 15 chromosome loci.
